# The stromal genome heterogeneity between breast and prostate tumors revealed by a comparative transcriptomic analysis

**DOI:** 10.18632/oncotarget.3478

**Published:** 2015-03-04

**Authors:** Kan He, Wenwen Lv, Dongni Zheng, Fei Cheng, Tao Zhou, Shoudong Ye, Qian Ban, Qilong Ying, Bei Huang, Lei Chen, Guohua Wu, Dahai Liu

**Affiliations:** ^1^ Center for Stem Cell and Translational Medicine, School of Life Sciences, Anhui University, Hefei City, Anhui, China; ^2^ Eli and Edythe Broad Center for Regenerative Medicine and Stem Cell Research, Department of Cell and Neurobiology, Keck School of Medicine, University of Southern California, Los Angeles, California, USA; ^3^ Department of Molecular Genetics, Shanghai Medical School, Fudan University, Shanghai, China; ^4^ Laboratory of Quality & Safety Risk Assessment for Sericultural Products and Edible Insects, Ministry of Agriculture, College of Biotechnology and Sericultural Research Institute, Jiangsu University of Science and Technology, Zhenjiang, China

**Keywords:** heterogeneity, breast tumor, prostate tumor, GSEA, pathway

## Abstract

Stromal microenvironment increases tumor cell survival, proliferation and migration, and promotes angiogenesis. In order to provide comprehensive information on the stromal heterogeneity of diverse tumors, here we employed the microarray datasets of human invasive breast and prostate cancer-associated stromals and applied Gene Set Enrichment Analysis (GSEA) to compare the gene expression profiles between them. As a result, 8 up-regulated pathways and 73 down-regulated pathways were identified in the breast tumor stroma, while 32 up-regulated pathways and 18 down-regulated pathways were identified in the prostate tumor stroma. Only 9 pathways such as tryptophan metabolism were commonly up or down regulated, but most of them (including ABC transporters) were specific for these two tumors. Several essential tumors stromal marker genes were also significantly identified. For example, CDH3 was significantly up-regulated in the stromals of both breast and prostate tumors, however EGFR was only significantly down-regulated in the stromal of breast tumor. Our study would be helpful for future therapeutic and predictive applications in breast and prostate cancers.

## INTRODUCTION

Cancer is not a single disease but includes numerous subtypes, each of which has its distinct histopathological and biological features [1]. Cancer heterogeneity refers to the distinction among different cancer cells in the aspects of morphology, phenotype and function, which includes the diversities in cellular morphology, gene expression, metabolism, motility, proliferation, and metastatic potential [2, 3]. The extensive heterogeneity exists both between tumors (inter-tumor heterogeneity) and within tumors (intra-tumor heterogeneity) [4]. The heterogeneity of cancer cells has been recognized as early as 1930s when the experiment showed that only a part of mouse tumors cells could give rise to new tumors when transplanted [5].

Although certain genetic similarities are shared between the primary and metastatic tumor cells, there are also some additional mutations in metastases. For both prostate cancer [6, 7] and breast cancer [8, 9], a number of mutations had been found which are different between the primary tumors and metastases. It suggests the presence of certain genetic diversity between them, and also reveals the evolution of tumor heterogeneity. A recent study analyzed primary tumors and metastases of invasive lobular breast cancer at single nucleotide resolution, which revealed mutations that only occur in metastases, and more metastasis loci lead to greater amount of mutations [10].

It is now widely recognized that cancer progression is not exclusively regulated by intra-tumor heterogeneity but also depends on the heterogeneity of tumor microenvironment, in which the stroma compartment plays an important role [11]. This suggests that understanding cancer by tumor cell genomic analysis is not sufficient, while analyzing tumor cells together with stromal cells may provide more comprehensive and meaningful data [12].

The tumor stroma is the complex arrangement of various stromal cells and extracellular matrix, which act in a coordinated manner to regulate cell function and maintain overall tissue homeostasis. In order to maintain homeostasis, the host tissue stroma interacts with carcinoma in a process similar to the tissue remodeling in wound repair [11, 13]. This process builds a new tumorigenesis-promoting stromal microenvironment, which supports tumor cell survival, proliferation and migration, and promotes angiogenesis [14].

One important reason why gene expression of cancer stroma has potential for cancer treatment and prediction is that cancer stroma maintains a normal genotype, because stromal reactions in response to cancer epithelium mostly only alter gene expression, without drastic and stochastic genomic changes [15, 16]. In addition, due to coevolution of cancer and stroma in the process of cancer progression, many genes were commonly expressed in stroma and cancer cells [17]. Active tumor evolution leads to therapeutic resistance, which is illustrative of dynamic interplay between tumor cells and their microenvironments when the selective pressure of drug therapy is applied. Despite obtained success in targeting tumor microenvironment, signifi­cant challenges still lie ahead for the implementation of stromal targeting in clinical practice [18]. Both the composition of tumor stroma and the stromal response to cancer are heterogeneous, while one stromal molecular signature correlates positively with survival in one tumor type, it may correlate inversely with survival of another tumor type, or it may be irrelevant at all. This heterogeneity leads to the complexity in clinical application of stromal molecular signatures and further studies of the stromal heterogeneity need to be done.

In the present study, we employed and reanalyzed the published microarray datasets of human breast and prostate cancer stroma from the public database library of GEO. Gene Set Enrichment Analysis (GSEA) was applied to compare the gene expression profiles between these two different invasive cancer types, especially in an effort to indicate the degree of stromal heterogeneity between them and identify candidate stromal gene expression signatures relevant to cancer progression. GSEA has the advantage to highlight genes weakly connected to the phenotype through pathway analysis which may be difficult to detect by using classical univariate statistics [19-23]. Through GSEA analysis, the critical pathways that were up- or down-regulated in the stroma of two types of cancer were identified, and we then constructed coexpression networks of related pathways with the signiﬁcantly core genes and transcription factors. Our study would be helpful for future therapeutic and predictive applications in breast and prostate cancer.

## RESULTS AND DISCUSSION

### Comparison of results between GSEA and DEGA

**Figure 1:**
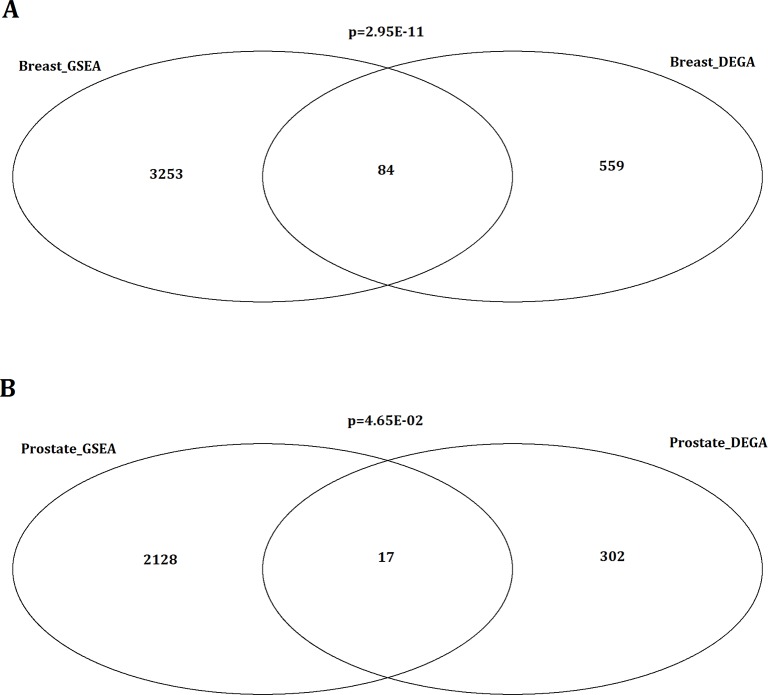
Comparison of stromal related genes between DEGA and GSEA results for breast tumor and prostate tumor (A) Venn diagram showing the overlapping significantly identified genes by the comparison of stromal related genes between DEGA and GSEA results for breast cancer. By comparison, there were 84 common genes between DEGA and GSEA results for breast tumor stromal. The significance of overlapping was p=2.95E-11. (B) Comparison of stromal related genes between DEGA and GSEA results for prostate cancer. Venn diagram showing the overlapping significantly identified genes by the comparison of stromal related genes between DEGA and GSEA results for prostate cancer. For prostate tumor, the common genes number was 17 with the overlapping significance of p=4.65E-02.

According to the approach of differentially expressed gene analysis (DEGA) studied by Planche et al., 643 and 319 differentially expressed genes between tumor and normal stroma were identified for breast cancer and prostate cancer, respectively (shown in Supplementary File 1) [24]. The DEGA approach was based on a paired analysis of differential expression using the package of limma with the cutoff of false positive rate (FDR) as 0.01. Here, we used standardized microarray preprocessing and GSEA with comprehensive expression profiles in order to find greater data convergence and provide a systematic insight into the associated pathways in both human breast and prostate tumor stromals. In our study, 8 up-regulated and 73 down-regulated pathways were significantly identified in breast tumor stromal, while 32 up-regulated and 18 down-regulated pathways were significantly identified in prostate tumor stromal based on the approach of GSEA. Totally, 3337 and 2145 genes were dysregulated in breast and prostate tumor stromal, respectively (shown in Supplementary File 2). By comparison, there were 84 common genes between DEGA and GSEA results for breast tumor stromal. The significance of overlapping was p=2.95E-11 (Figure 1A). For prostate tumor, the common genes number was 17 with the overlapping significance of p=4.65E-02 (Figure 1B). It indicated that our GSEA results would be not only consistent with the previous DEGA results but also more comprehensive. In addition, 360 and 342 dysregulated transcription factors (TFs) were further identified in breast tumor and prostate tumor stromal, respectively (shown in Supplementary File 3).

### The stromal genome heterogeneity in multiple pathways level

**Figure 2:**
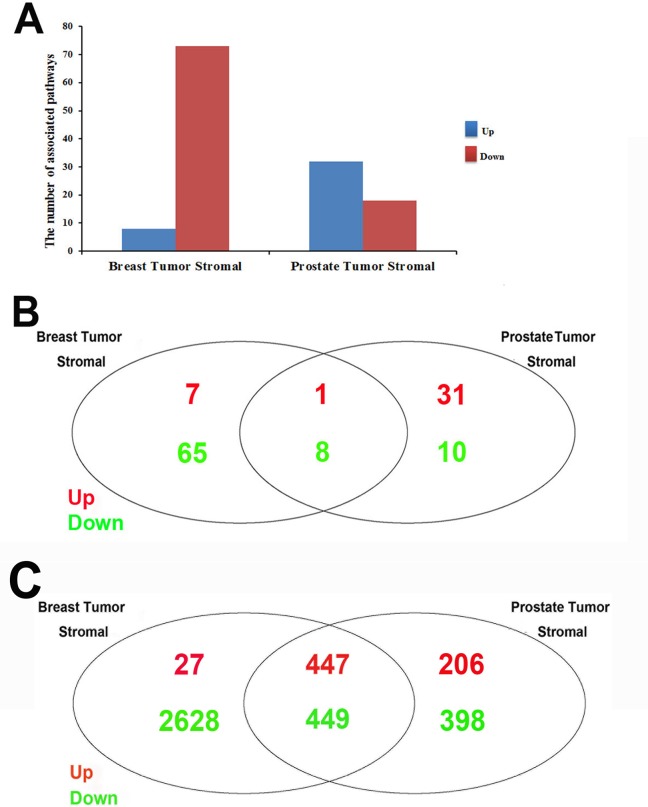
Comparisons of GSEA results between breast tumor and prostate tumor stromals (A) The number of identified up-or downregulated pathways in stromal regions of human breast and prostate cancer. The bar chart showing the number of significantly identified pathways (P≤0.01) in stromal regions of human breast and prostate cancer. The data was shown in Supplementary File 2. X-axis represents our studies; Y-axis represents the number of significantly identified pathways. Blue color (up) is for upregulated pathways and red color (down) is for downregulated pathways. There were 8 and 73 in study of human breast cancer, the study in stromal regions of human breast cancer in GSE26910; 32 and 18 in study of human prostate cancer, the study in stromal regions of human prostate cancer, respectively, for up-and down-regulated pathways. (B) Comparison of stromal related pathways between human breast and prostate cancer. Venn diagram showing the overlapping significantly identified pathways by the comparison of stromal related pathways between human breast and prostate cancer. Pathways of P-value less than 0.01 were considered to be significantly regulated. Red color is for up-regulated pathways and green color is for down-regulated pathways. There were 1 up-regulated and 8 down-regulated stromal related pathways in common between human breast and prostate cancer. (C) Comparison of stromal related genes between human breast and prostate cancer. Venn diagram showing the overlapping significantly identified genes by the comparison of stroma related genes between human breast and prostate cancer. Genes of p-value less than 0.01 were considered to be significantly regulated. Red color is for up-regulated genes and green color is for down-regulated genes. There were 447 up-regulated and 449 down-regulated stromal related genes in common between human breast and prostate cancer.

**Figure 3:**
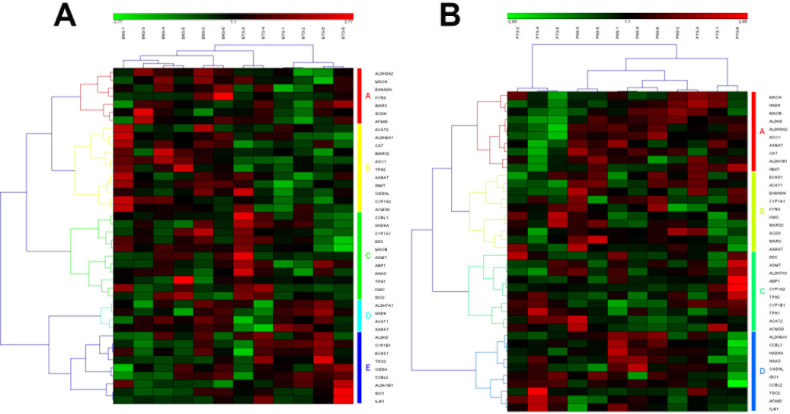
The heat map and hierarchical clustering in Tryptophan metabolism pathway (A) It showed the heat map and hierarchical clustering in Tryptophan metabolism pathway from human breast tumor stromal. There were 42 involved genes in Tryptophan metabolism pathway from human breast tumor stromal, which were clustered into 5 groups (the group from A to E). (B) It showed the heat map and hierarchical clustering in Tryptophan metabolism pathway from human prostate tumor stromal. There were 40 involved genes in Tryptophan metabolism pathway from human prostate tumor stromal, which were clustered into 4 groups (the group from A to D). Red is for up-regulated and green is for down-regulated.

Firstly, the common GSEA method was applied to the stromal regions of human breast and prostate tumors. For individual analysis, we obtained the significant pathways in each dataset, which were summarized in Figure 2A and Supplementary File 2. Firstly, we compared the up-regulated and down-regulated pathways in the stromals of both tumor types, respectively. Interestingly, 9 highly common pathways were identified, including only 1 up-regulated and 8 down-regulated pathways common to the breast tumor and prostate tumor stromal (shown in Table 1 and Figure 2B). The most down-regulated pathways were metabolism related pathway, such as amino sugar and nucleotide sugar metabolism, riboflavin metabolism, mucin type O-glycan biosynthesis, and glycosphingolipid biosynthesis-lacto and neolacto series identified in both breast and prostate tumor stromal [29-31]. Besides, cell adhesion molecules (CAMs) are related to environmental information processing, which have a crucial role in tumor progression, in particular during invasion and metastasis [32]. The pathway of leukocyte transendothelial migration is associated with organismal systems. The pathway of phagosome is cellular processes related. Bacterial invasion of epithelial cells, one part of human diseases, which has reported that induction of inflammation by bacteria and viral infections increases cancer risk [33]. The only one upregulated pathway was tryptophan metabolism, which is metabolism related and altered in patients suffering from gynecological cancer compared to healthy controls (shown in Figure 3) [34].

**Table 1:** The dysregulated pathways identified in both human breast and prostate tumor stromal

Regulation	Pathways	Groups
Commonly up-regulated (1)	00380: Tryptophan metabolism	Metabolism
Commonly down-regulated(8)	04514: Cell adhesion molecules (CAMs)	Environmental Information Processing
	00512: Mucin type O-Glycan biosynthesis	Metabolism
	04670: Leukocyte transendothelial migration	Organismal Systems
	00740: Riboflavin metabolism	Metabolism
	04145: Phagosome	Cellular Processes
	00601: Glycosphingolipid biosynthesis - lacto and neolacto series	Metabolism
	00520: Amino sugar and nucleotide sugar metabolism	Metabolism
	05100: Bacterial invasion of epithelial cells	Human Diseases
Up-regulated in BTS and down-regulated in PTS (2)	02010: ABC transporters	Environmental Information Processing
	04742: Taste transduction	Organismal Systems
Down-regulated in BTS and up-regulated in PTS (10)	00250: Alanine, aspartate and glutamate metabolism	Metabolism
	04110: Cell cycle	Cellular Processes
	00860: Porphyrin and chlorophyll metabolism	Metabolism
	00670: One carbon pool by folate	Metabolism
	05012: Parkinson's disease	Human Diseases
	00190: Oxidative phosphorylation	Metabolism
	03020: RNA polymerase	Genetic Information Processing
	00450: Selenocompound metabolism	Metabolism
	03030: DNA replication	Genetic Information Processing
	03022: Basal transcription factors	Genetic Information Processing

9 highly common pathways were identified, including only 1 up-regulated and 8 down-regulated pathways common to the breast tumor and prostate tumor stromal. 2 pathways were up-regulated in breast tumor stromal and down-regulated in prostate tumor stromal, but 10 pathways were down-regulated in breast tumor stromal and up-regulated in prostate tumor stromal.

**Figure 4:**
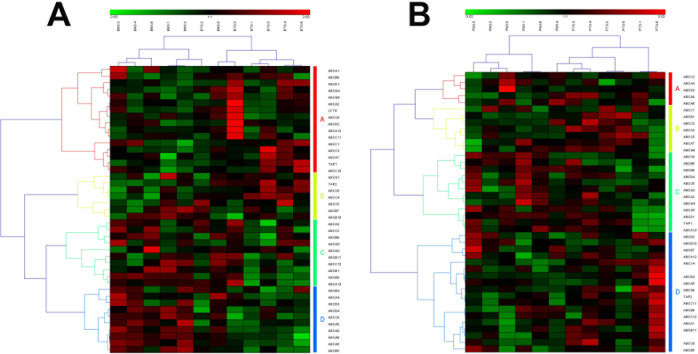
The heat map and hierarchical clustering in ABC transporters pathway (A) It showed the heat map and hierarchical clustering in ABC transporters pathway from human breast tumor stromal. There were 56 involved genes in ABC transporters pathway from human breast tumor stromal, which were clustered into 4 groups (the group from A to D). (B) It showed the heat map and hierarchical clustering in ABC transporters pathway from human prostate tumor stromal. There were 42 involved genes in ABC transporters pathway from human prostate tumor stromal, which were mainly clustered into 4 groups (the group from A to D). Red is for up-regulated and green is for down-regulated.

In addition, there were several tissue-specially identified pathways. 2 pathways were up-regulated in breast tumor stromal and down-regulated in prostate tumor stromal, such as the pathways of taste transduction and ABC transporters (Table 1). ATP-binding cassette (ABC) transporters are a family of transporter proteins that contribute to drug resistance via ATP-dependent drug efflux pumps [35]. Recent studies suggested that many ABC transporter superfamily members are highly expressed in breast cancer, which could be molecular target for the treatment of breast cancer [36]. The pathway of ABC transporters in human prostate cancer has little reported (shown in Figure 4).

Moreover, 10 pathways were down-regulated in breast tumor stromal and up-regulated in prostate tumor stromal, such as oxidative phosphorylation. Cancer is a heterogeneous class of diseases, each of which has its own metabolic characteristics, even if each of the tumor, which includes different cell constituted a difference in the mode of metabolism [37]. Owing to differences in tumor size, hypoxia, and the sequence of oncogenes activated, some studies illustrate a reduction of oxidative phosphorylation (OXPHOS) capacity in different types of cancer cells, other investigations revealed contradictory modifications with the upregulation of OXPHOS components and a larger dependency of cancer cells on oxidative energy substrates for anabolism and energy production [38]. Our research found that Oxidative phosphorylation is down-regulated in breast tumor stromal and up-regulated in prostate tumor stromal.

### The stromal genome heterogeneity in the levels of target gene and TFs

**Figure 5:**
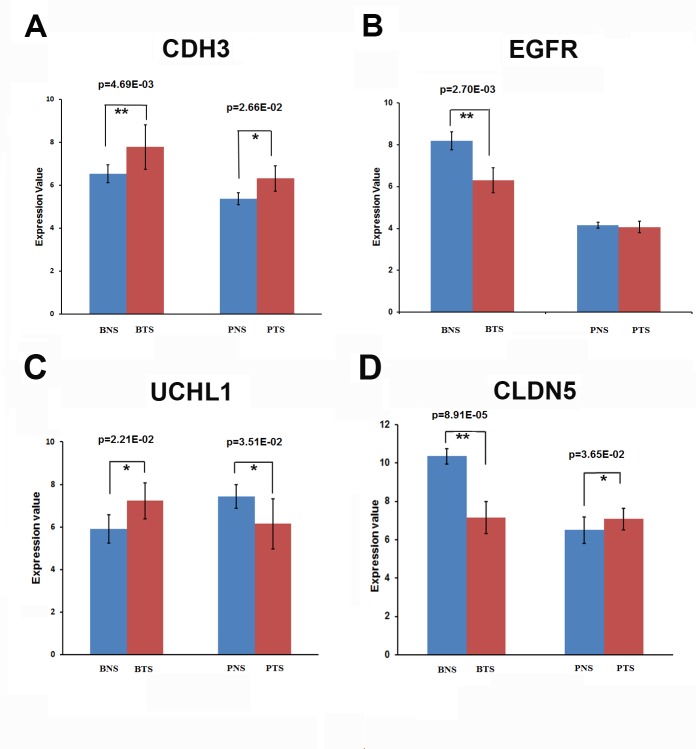
The expression patterns of some target genes for breast and prostate tumor stromals (A) The expression of CDH3 gene was significantly up-regulated both in breast tumor stromal (BTS) (p=4.69E-03) and in prostate tumor stromal (PTS) (p=2.66E-02) compared to the normal stromals (BNS or PNS). (B) The expression of EGFR was significantly down-regulated in breast tumor stromal (BTS) (p=2.70E-03), but remained the same level in prostate tumor stromal (PTS) compared to the normal stromals (BNS or PNS). (C) The expression patterns of UCHL1. The expression of UCHL1 was significantly up-regulated in breast tumor stromal (BTS) (p=2.21E-02), but was significantly down-regulated in prostate tumor stromal (PTS) (p=3.51E-02) compared to the normal stromals (BNS or PNS). (D) The expression patterns of CLDN5. In breast tumor stromal (BTS), the expression of CLDN5 was significantly down-regulated (p=8.91E-05), but was significantly up-regulated in prostate tumor stromal (PTS) (p=3.65E-02) compared to the normal stromals (BNS or PNS).

Our analysis revealed that 447 up-regulated genes and 449 down-regulated genes were common in the stromals of both tumor types, which were shown in Figure 2C. for individual analysis, we obtained the significant TFs in each dataset, which were summarized in Supplementary File 3. Furthermore, our research also found that some specific genes and transcriptional factors were heterogeneously regulated in breast and prostate tumor stromal, such as CDH3, EGFR, UCHL1 and CLDN. The expression patterns of these target genes were shown in Figure 5.

P-cadherin, a classical cadherin encoded by the CDH3 gene [39], are engaged in various cellular activities including motility, invasion, and signaling of tumor cells, in addition to cell adhesion. Regarding other classic cadherins, placental (P)-cadherin was first shown in mouse placenta [40], in humans its expression is not identified in placenta but is present in a few organs such as mammary gland and prostate [41]. In breast cancer, P-cadherin is frequently overexpressed in high-grade tumours and is extensively associated with tumour aggressiveness and poor patient prognosis [42]. In prostate cancer, CDH3 is an important cell-cell adhesion molecule and is a prostate cancer susceptibility candidate gene [43]. Expression of the CDH3 gene in the tumor cell compartment of prostate and breast cancer samples and its increase with the degree of tumor progression were accordant with its implication in tumor development [44]. Surprisingly, whereas most researches have reported its expression to be limited to the tumor cell compartment, CDH3 gene was also identified to expression in stromal compartments of invasive cancers in our study. The expression of CDH3 gene was significantly up-regulated both in breast tumor stromal (p= 4.69E-03) and in prostate tumor stromal (p=2.66E-02) (Figure 5A). Elucidation of the role of CDH3 in the tumor stromal will be of interest. It is attractive to speculate, for example, that CDH3-positive stromal cells reflect an active state that may contribute to tumor aggressiveness.

It has been long established that the epidermal growth factor receptor (EGFR) and the EGF-family of peptide growth factor play an important role in the pathogenesis and progression of different carcinoma types. The proteins of EGF ligand/receptor system are found to be frequently expressed in the majority of human carcinomas. In this study, EGFR was identified to be heterogeneously regulated in breast and prostate cancer. The expression of EGFR was significantly down-regulated in breast tumor stromal (p=2.70E-03), but remained the same level in prostate tumor stromal compared to the normal stromal (Figure 5B). The binding of EGF to EGFR leads to the phosphorylation of EGFR, which then stimulates the signaling pathways that promoting cell proliferation, adhesion, and resistance to apoptosis. It has been showed that EGFR signaling regulates angiogenesis both directly and indirectly [45]. Tumor progression is a complex process that involves the interaction of tumor cells with surrounding stromal. Almost all types of cells in stromal have the expression of EGFR. Therefore, when studying the effect of EGFR signaling in cancer progression, it is important that we take into consideration of EGFR signaling not only in tumor cells but also in surrounding stromal cell populations. This may accounts for some failures in targeting EGFR signaling only in tumor cells without considering the stromal EGFR regulation. Even in tumors with EGFR-independent growth, EGFR signaling in the tumor stromal may indirectly promote tumor progression by mediating complex interactions between tumor and stomal [46].

The critical roles played by protein ubiquitination in various biological processes including cell proliferation, cell cycle, apoptosis, signal transduction, while its deregulation contributes to tumor initiation and progression [47, 48]. Ubiquitin carboxyl-terminal esterase L1 (UCHL1) is a member of a gene family whose products transfer ubiquitin directly to protein substrates and release ubiquitin from tandemly conjugated ubiquitin monomers [49, 50]. Deregulation of UCHL1 has been observed in solid tumors such as pancreatic cancer [51], non-small cell lung cancer [52], colorectal cancer [53], osteosarcoma [54], and oesophageal cancer [55]. Expression profiling data from various tumor types demonstrated that UCHL1 is either up- or downregulated owing to promoter hypo- or hypermethylation depending on the type of malignant tissue. In this research, UCHL1 was identified to be heterogeneously regulated in breast and prostate tumor stromal. The expression of UCHL1 was significantly up-regulated in breast tumor stromal (p=2.21E-02), but was significantly down-regulated in prostate tumor stromal (p=3.51E-02) compared to the normal stromals (Figure 5C).

The claudin (CLDN) genes encode a family of highly related proteins important in tight junction formation and function. Recently, it has become obvious that CLDN gene expression is frequently altered in various cancers [56, 57]. Specifically, CLDN1,3,4,5,7,10,16 have been found altered in several human tumors [56]. The first vascular-specific claudin identified was CLDN5, also known as transmembrane protein deleted in velocardiofacial syndrome (TMVCF) [58, 59]. Recent research reported that CLDN5 was highly expressed in vascular endothelial cells, suggesting a new target for antiangiogenic therapy [60]. Our research indicates that, depending on the type of neoplasia, CLDN5 may be diminished, elevated or mislocated in tumor stromal compared to normal stromal. In breast tumor stromal, the expression of CLDN5 was significantly down-regulated (p=8.91E-05), but was significantly up-regulated in prostate tumor stromal (p=3.65E-02) compared to the normal stromals (Figure 5D). Overall, a better knowledge of claudin expression in normal and neoplastic tissues may have applications in the detection, prognosis and therapy of several human cancers.

## METHODS

### Microarray data collection and preprocessing

The gene expression proﬁling studies related to stromal regions of human breast and prostate cancer were searched in GEO (www.ncbi.nlm.nih.gov/geo/). Data sets were reanalyzed if they met the following conditions: (1) the data were genome-wide, (2) comparison was conducted in the stromal regions between breast tumors and prostate tumors, and (3) complete microarray raw or normalized data were available. The data set of GSE26910, contributed by Paolo Provero, was finally chosen for our re-analysis [24]. In this data set, a total of 24 RNA samples were tested for RNA quality and each of the 24 sample targets was hybridized to Affymetrix Human Genome U133 Plus2.0 GeneChip arrays. There were six biological replicates for prostate normal stromals (GSM662756, GSM662758, GSM662760, GSM662762, GSM662764, GSM662766, the group named as PNS), six for prostate tumor stromals (GSM662757, GSM662759, GSM662761, GSM662763, GSM662765, GSM662767, the group named as PTS), six for breast normal stromals (GSM662768, GSM662770, GSM662772, GSM662774, GSM662776, GSM662778, the group named as BNS) and six for breast tumor stromals (GSM662769, GSM662771, GSM662773, GSM662775, GSM662777, GSM662779, the group named as BTS).

For the purpose of evaluating the effect of reprocessing on the comparison, the data were reprocessed using software packages developed in version 2.6.0 of Bioconductor and R version 2.10.1 [25]. Each Affymetrix data set was background-adjusted and normalized and log2 probe-set intensities were calculated using the Robust Multichip Averaging (RMA) algorithm in the Affy package [26].

### Gene set enrichment analysis

Here, our gene set enrichment analysis was performed on each study above to identify signiﬁcantly related pathways and genes to either stromal regions of breast cancer or stromal regions of prostate cancer by using Category package in Bioconductor ver. 2.6.0 [27]. GSEA is aimed at determining whether the members of a gene set S are randomly spread throughout the entire reference gene list L or are found primarily at the top or bottom of L. One of the advantages of GSEA is the relative robustness to noise and outliers in the data. In our research, the gene sets showed by less than 10 genes were excluded. The t-statistic mean of the genes was computed in each KEGG (Kyoto Encyclopedia of Genes and Genomes) pathway. Using a permutation test with 1,000 times, 0.01 was chosen as the signiﬁcance level p values of the cutoff for the most signiﬁcant pathways related to stromal tumor. Consequently, the signiﬁcant pathways and genes between tumor and normal were indicated in breast or prostate. Subsequently, based on the datasets between in breast cancer and in prostate cancer, the comparison of GSEA results was performed to demonstrate the regulatory mechanisms of gene expression by stromal regions of each other. The following classiﬁcation of identiﬁed pathways was based on the KEGG pathway map br08901 of BRITE Functional Hierarchies in the database of KEGG (http://www.genome.jp/kegg-bin/get_htext?br08901.keg). The annotation of signiﬁcant genes in each pathway was performed by using the biomaRt package (http://www.biomart.org/) BioMart ver. 0.8 rc3 (version 0.8 of release candidate 3). Next, for each signiﬁcant pathway, through hierarchical clustering with Euclidean distance, clustering on groups or genes was performed based on the identiﬁed genes' expression.

### Regulatory elements and transcription factors of coregulated genes

A web server called DiRE (Distant Regulatory Elements of coexpressed genes, http://dire.dcode.org/) were also used, which is based on the Enhancer Identiﬁcation (EI) method, to predict common regulatory elements (REs) for our input genes that have a cofunction in each identiﬁed signiﬁcantly related pathway [28]. It predicts function-speciﬁc REs consisting of clusters of speciﬁcally associated transcription factor binding sites (TFBSs), and scores the association of individual transcription factors (TFs) with the biological function shared by the group of input genes. We selected a random set of 5000 genes in the genome of homo sapiens as the source of background genes. There were two major parameters of our predicted TFs: (1) TF occurrence for the percentage of candidate regulatory elements containing a conserved binding site for a particular TF and (2) TF importance for the product of TF occurrence and TF weight. To be included in our candidate associated TFs with input gene sets, the value of TF importance should be more than 0.05.

## SUPPLEMENTARY MATERIALS AND TABLES






